# Assessment of the degree of adherence of medical laboratories to KDIGO 2012 guideline for evaluation and management of CKD in Czechia and Slovakia

**DOI:** 10.11613/BM.2019.030704

**Published:** 2019-08-05

**Authors:** Šálek Tomáš, Friedecký Bedřich, Kratochvíla Josef, Pelinková Květa, Budina Marek

**Affiliations:** 1 Department of Clinical Biochemistry and Pharmacology, Tomas Bata Hospital, Zlín, Czech Republic; 2 Department of Biomedical Sciences, Medical Faculty of the University of Ostrava, Ostrava - Zábřeh, Czech Republic; 3 SEKK, spol. s.r.o., Pardubice, Czech Republic; 4 Institute of Medical Biochemistry and Laboratory Diagnostics, General University Hospital and The First Faculty of Medicine of Charles University in Prague, Prague, Czech Republic

**Keywords:** creatinine, cystatin C, glomerular filtration, estimated glomerular filtration rate, post-analytical phase

## Abstract

**Introduction:**

The aim of the study is to assess the degree of adherence of medical laboratories to Kidney Disease Improving Global Outcomes (KDIGO) 2012 Clinical Practice Guideline for the Evaluation and Management of Chronic Kidney Disease (CKD) in laboratory practice in Czechia and Slovakia.

**Materials and methods:**

An electronic questionnaire on adherence to KDIGO 2012 guideline was designed by an external quality assessment (EQA) provider SEKK spol. s.r.o. The questionnaire was placed and distributed through website to all medical biochemistry laboratories in Czechia and Slovakia (N = 396).

**Results:**

A total of 212 out of 396 laboratories responded to the questions, though some laboratories only answered some questions, those applicable to their practice. A total of 48 out of 212 laboratories adopted the KDIGO 2012 guideline in full extent. The metrological traceability of creatinine measurement to standard reference material of SRM 967 was declared by 180 out of 210 laboratories (two of the responding laboratories did not measure creatinine). Thirty laboratories are not well educated on traceability of creatinine measurement and seven laboratories do not calculate estimated glomerular filtration rate (eGFR). Both urinary albumin concentration and albumin to creatinine ratio are reported by 144 out of 175 laboratories (37 of the responding laboratories did not measure urinary albumin).

**Conclusion:**

Majority of laboratories in Czechia and Slovakia adopted some parts of the KDIGO 2012 guideline in their practice, but only 23% of the laboratories apply them completely. Thus, further education and action should be conducted to improve its implementation.

## Introduction

The current definition of chronic kidney disease (CKD) includes an estimated glomerular filtration rate (eGFR) decreased below 60 mL/min/1.73m^2^ and/or the presence of kidney damage for a time period longer than 3 months with implications for health (*1*). The overall prevalence of CKD was 11.6% in China and 12.9% in the United States (*2*). The accurate prevalence of CKD in Czechia and Slovakia is not known. In Europe, the adjusted CKD stages 1–5 prevalence varies between 3.31% in Norway and 17.3% in northeast Germany (*3*). The risk factors for development of CKD are defined. They are: age, hypertension, diabetes, obesity, cardiovascular disease and others. Simple obesity increases the risk of CKD independently of dyslipidaemia, diabetes, and hypertension (*4*). Correct diagnosis of CKD strongly depends on serum creatinine, cystatin C and urinary albumin measurement. Albuminuria usually precedes the decline of kidney function (*5*). Clinical laboratories are recommended to measure creatinine by enzymatic method traceable to standard reference material SRM 967 (*6*). Cystatin C should be measured with traceability of measurement to international reference material DA ERM 471 (*7*). Patients with CKD are at high or very high cardiovascular risk and have lower low density lipoprotein (LDL) cholesterol treatment targets (*8*). Identification of these high risk patients is the main reason why Kidney Disease Improving Global Outcomes (KDIGO) 2012 guideline should be implemented. The Czech Society for Clinical Biochemistry and Czech Nephrology Society created Guidelines on diagnostics of Chronic Kidney Disease in 2014. Key points of KDIGO 2012 guidelines are included but the unit of eGFR is changed from mL/min to mL/s (*9*). The intention of this guideline was to harmonize creatinine and cystatin C measuring and eGFR reporting. The guideline strongly recommends using standardized creatinine and cystatin C methods in laboratories. Many articles in this field were published in Czech national journal of Clinical Biochemistry and Metabolism. This topic was also repeatedly discussed by external quality assessment (EQA) supervisors in EQA reports. Before the publication of these guidelines there was lower level of metrological traceability of creatinine measurement and the modification of diet in renal disease (MDRD) equation was the most common equation for estimated glomerular filtration rate from serum creatinine (eGFRcrea). Patient safety, improvement of the level of standardization of measurement and improvement of the harmonization of reporting of laboratory test results are reasons why we performed this survey. The aim of the study is to assess the degree of adherence of medical laboratories to KDIGO 2012 Clinical Practice Guideline for the Evaluation and Management of CKD in laboratory practice in Czechia and Slovakia.

## Materials and methods

### Study design

An electronic questionnaire on adherence to KDIGO 2012 guideline was designed by an EQA provider SEKK spol. s.r.o., with registered office in Pardubice, Czechia. The questionnaire was placed and distributed through EQA SEKK’s website www.sekk.cz and sent by e-mail to all medical biochemistry laboratories in Czechia and Slovakia (N = 396) as a part of the basic clinical chemistry EQA scheme. The questions of the questionnaire on adherence to KDIGO 2012 guideline in Czechia and Slovakia are shown in Table 1. Information on the survey and instructions for completing the questionnaire were part of the EQA scheme. Laboratory directors had responsibility for data entry and data were collected in the period from January 7^th^ to January 25^th^ 2019. The entry of results could be monitored by a link to the website. Participation in the survey was voluntary. In addition to the questionnaire, data on the type of creatinine method used among laboratories were collected from the results of first basic clinical chemistry EQA scheme (the number of participants was 188). Information on the type of creatinine method was not derived from the survey. These data were collected as a creatinine enzymatic method is recommended by KDIGO 2012 guideline. In the end, 212 laboratories participated in the study. All data were collected and no laboratory response was excluded. We did not compare results among different kinds of laboratories. The ideal target is full guideline implementation. Full guideline implementation was defined as the adoption of the KDIGO 2012 guideline in full extent including cystatin C traceable method and eGFR. Partial guideline implementation was defined as creatinine measurement traceable to standard reference material SRM 967. After the survey had been completed, all participants received interpretative comments from the EQA supervisors, containing references to related pages of the KDIGO 2012 guideline. These comments were not part of the questionnaire.

**Table 1:** The list of questions of the questionnaire on adherence to KDIGO 2012 guideline among laboratories in Czechia and Slovakia

**Question**	**Possible answers**
**1. Do you measure serum creatinine concentrations?**	No Yes
**2. Do you perform serum cystatin C measurement?**	No Yes
**3. Do you measure albumin in urine?**	No Yes
**4. Do you calculate eGFR? (3 options possible)**	No From serum creatinine From serum cystatin C Combined estimation from serum creatinine and cystatin C
**5. What information on metrological traceability does the manufacturer of your creatinine method provide?**	Traceability to SRM 967 Traceability is not declared Information is not available
**6. Which equation do you use for eGFRcrea?**	CKD-EPI from 2009 MDRD equation Another equation (provide)
**7. In which subgroups of patients do you report eGFRcrea?**	In all patients On request
**8. In which subgroups of patients do you not report eGFRcrea?**	Acute care Oncology patients Pregnant women Another subgroup (provide information)
**9. What decision limit of eGFRcrea do you use?**	60 mL/min 90 mL/min Another (provide information)
**10. What information on metrological traceability does the manufacturer of your cystatin C method provide?**	Traceability to international reference material DA ERM 471 Traceability is not declared Information is not available
**11. Which equation do you use for eGFRcys?**	CKD-EPI from 2012 CAPA equation Another equation (provide)
**12. In which subgroups of patients do you report eGFRcys?**	In all patients On request
**13. In which subgroups of patients do you not report eGFRcys?**	provide information
**14. How do you calculate eGFR?**	By laboratory information system By web calculator (specify) By excel We use different way (provide information)
**15. Which information do you report when you determine albuminuria?**	Only urinary albumin concentration Only albumin to creatinine ratio Both urinary albumin concentration and albumin to creatinine ratio We use different procedure (describe)
**16. Which way for albuminuria determination prevails in your laboratory?**	Spot urine samples Urine timed collection
**17. How many decimal places do you use for creatinine concentration reporting in µmol/L?**	We do not control it, it depends on laboratory information system Whole numbers 1 decimal place 2 decimal places It depends on uncertainty of measurement
**18. How many decimal places do you use for cystatin C concentration reporting in mg/L?**	We do not control it, it depends on laboratory information system 1 decimal place 2 decimal places It depends on uncertainty of measurement
KIDGO – Kidney Disease Improving Global Outcomes. eGFR - estimated glomerular filtration rate. SRM - standard reference material. CKD-EPI - chronic kidney disease epidemiology collaboration. eGFRcrea - glomerular filtration rate estimated from serum creatinine. MDRD - modification of diet in renal disease. eGFRcys - glomerular filtration rate estimated from serum cystatin C. CAPA - Caucasian, Asian, Paediatric, Adult.	

### Statistical analysis

Data were collected to Microsoft Excel Office 2007 program (Microsoft, Washington, USA). The total absolute number of specific responses of each specific question and their relative percentages compared to the total number of responses to the particular question were calculated. The denominator of the percentages for full and partial guideline implementation was the total number of participants of the survey (N = 212).

## Results

The response rate to the questionnaire was 54% (212 out of 396 laboratories). The answers to the questionnaire by laboratory participants on adherence to KDIGO 2012 guideline are provided in Table 2. Of all participants, 210 laboratories measured serum creatinine, 57 serum cystatin C, and 175 measured urinary albumin. A total of 53% (99/188) laboratories measured creatinine by enzymatic method. The Jaffe method was measured by 89 laboratories. The Chronic Kidney Disease Epidemiology Collaboration (CKD-EPI) equation was used by 75% of all laboratories which were calculating eGFRcrea. Both urinary albumin concentration and albumin to creatinine ratio are reported by 144 out of 175 laboratories (37 of the responding laboratories did not measure urinary albumin). A total of 48 out of 212 laboratories adopted the KDIGO 2012 guideline in full extent including cystatin C traceable method and eGFR. The partial KDIGO 2012 guideline implementation was found in 180 out of 212 (85%) laboratories. The interpretative comments provided by the EQA supervisors after the survey are shown in Table 3.

**Table 2:** Answers to the questionnaire by laboratory participants on adherence to KDIGO 2012 guideline

**Question**	**Total answers (N)**	**Answer – number of laboratories, N (%)**
**1. Do you measure serum creatinine concentrations?**	212	No – 2 (1) Yes – 210 (99)
**2. Do you perform serum cystatin C measurement?**	208	No – 151 (73) Yes – 57 (27)
**3. Do you measure albumin in urine?**	210	No – 35 (17) Yes – 175 (83)
**4. Do you calculate eGFR? (3 options possible)**	199	No – 7 (4) From serum creatinine – 192 (96) From serum cystatin C – 41 (21) Combined estimation from serum creatinine and cystatin C – 24 (12)
**5. What information on metrological traceability does the manufacturer of your creatinine method provide?**	195	Traceability to SRM 967 – 180 (92) Traceability is not declared – 8 (4) Information is not available – 7 (4)
**6. Which equation do you use for eGFRcrea?**	197	CKD-EPI from 2009 – 148 (75) MDRD equation – 42 (21) Another equation – 7 (4) (6x Lund-Malmö, combination of equations above)
**7. In which subgroups of patients do you report eGFRcrea?**	197	In all patients – 139 (71) On request – 58 (29)
**8. In which subgroups of patients do you not report eGFRcrea?**	84	Acute care – 7 (8) Oncology patients – 2 (3) Pregnant women – 21 (25) Another subgroup – 54 (64) (patients on dialysis, with creatinine above 250 umol/L)
**9. What decision limit of eGFRcrea do you use?**	176	60 mL/min – 64 (36) 90 mL/min – 100 (57) Another 12 (7) (9x 66 mL/min, 2x 120 mL/min, 1x 69 mL/min)
**10. What information on metrological traceability does the manufacturer of your cystatin C method provide?**	57	Traceability to international reference material ERM DA 471 – 51 (89) Traceability is declared only generally – 4 (7) Information is not available – 2 (4)
**11. Which equation do you use for eGFRcys?**	50	CKD-EPI from 2012 – 48 (96) CAPA – 1 (2) Another equation – 1 (2) (equation by Grubb)
**12. In which subgroups of patients do you report eGFRcys?**	51	In all patients – 42 (82) On request – 9 (18)
**13. In which subgroups of patients do you not report eGFRcys?**	3	Acute care – 0 (0) Oncology patients – 1 (33) Pregnant women – 2 (67)
**14. How do you calculate eGFR?**	186	By laboratory information system – 184 (99) By web calculator – 0 (0) By excel – 2 (1)
**15. Which information do you report when you determine albuminuria?**	175	Only urinary albumin concentration – 16 (9) Only albumin to creatinine ratio – 15 (9) Both urinary albumin concentration and albumin to creatinine ratio – 144 (82)
**16. Which way for albuminuria determination prevails in your laboratory?**	169	Spot urine samples – 155 (92) Urine timed collection – 14 (8)
**17. How many decimal places do you use for creatinine concentration reporting in µmol/L?**	198	We do not control it, it depends on laboratory information system – 13 (7) Whole numbers – 116 (59) 1 decimal place – 56 (28) 2 decimal places – 13 (7) It depends on uncertainty of measurement – 0 (0)
**18. How many decimal places do you use for cystatin C concentration reporting in mg/L?**	59	We do not control it, it depends on laboratory information system – 2 (3) 1 decimal place – 6 (10) 2 decimal places – 51 (86) It depends on uncertainty of measurement – 0 (0)
KIDGO – Kidney Disease Improving Global Outcomes. eGFR - estimated glomerular filtration rate. SRM - standard reference material. CKD-EPI - chronic kidney disease epidemiology collaboration. eGFRcrea - glomerular filtration rate estimated from serum creatinine. MDRD - modification of diet in renal disease. eGFRcys - glomerular filtration rate estimated from serum cystatin C. CAPA - Caucasian, Asian, Paediatric, Adult.		

**Table 3:** Interpretative comments by supervisors on questions related to KDIGO 2012 guideline adherence among laboratories in Czechia and Slovakia after the survey

**Question text**	**Educational comment – recommendation by KDIGO 2012 Clinical Practice Guideline and according to current knowledge**
**1. Do you measure serum creatinine concentrations?**	–
**2. Do you perform serum cystatin C measurement?**	–
**3. Do you measure albumin in urine?**	–
**4. Do you calculate eGFR? (3 options possible)**	KDIGO guideline recommends reporting eGFR together with concentration of creatinine and or cystatin C (page 7 of the KDIGO guideline).
**5. What information on metrological traceability does the manufacturer of your creatinine method provide?**	KDIGO guideline recommends method traceable to international standard reference material for measurement of serum creatinine (page 39 of the KDIGO guideline).
**6. Which equation do you use for eGFRcrea?**	KDIGO guideline recommends CKD-EPI equation from 2009 (page 7 of the KDIGO guideline), MDRD equation is validated only for patients with CKD.
**7. In which subgroups of patients do you report eGFRcrea?**	eGFRcrea was developed for hemodynamically stable non–acute patients without muscle loss. KDIGO guideline recommends eGFRcrea for initial assessment of GFR in most circumstances (page 38 of the KDIGO guideline).
**8. In which subgroups of patients do you not report eGFRcrea?**	No eGFRcrea equation was validated for pregnant women and acute care patients. But physicians still need to dose a drugs excreted by kidneys. KDIGO guideline recommends eGFRcys for confirmatory testing in specific circumstances when eGFR based on serum creatinine is less accurate (page 38 of the KDIGO guideline).
**9. What decision limit of eGFRcrea do you use?**	The limit of 60 mL/min defines CKD by GFR criterion The limit of 90 mL/min defines normal GFR (page 5 of the KDIGO guideline)
**10. What information on metrological traceability does the manufacturer of your cystatin C method provide?**	KDIGO guideline recommends standardized method traceable to DA471/IFCC for measurement of serum cystatin C (page 51 of the KDIGO guideline).
**11. Which equation do you use for eGFRcys?**	KDIGO guideline recommends CKD-EPI equation from 2012 (page 51 of the KDIGO guideline). The CAPA equation was also validated.
**12. In which subgroups of patients do you report eGFRcys?**	eGFRcys was developed for hemodynamically stable non–acute patients (page 50 of the KDIGO guideline).
**13. In which subgroups of patients do you not report eGFRcys?**	No eGFRcys equation was validated for pregnant women and acute care patients (page 50 of the KDIGO guideline.) But physicians still need to dose a drugs excreted by kidneys.
**14. How do you calculate eGFR?**	The calculation by laboratory information system is probably the most practical approach.
**15. Which information do you report when you determine albuminuria?**	The reporting of albumin to creatinine ratio in addition to albumin concentration is recommended by KDIGO guideline (page 56 of the KDIGO guideline).
**16. Which way for albuminuria determination prevails in your laboratory?**	The spot urine sample is probably an easier and more practical approach compared to urine timed collection with the risk of incomplete collections. Untimed urine samples are included in the KDIGO guideline (page 56 of the KDIGO guideline).
**17. How many decimal places do you use for creatinine concentration reporting in µmol/L?**	KDIGO guideline recommends that serum creatinine concentration should be rounded to the nearest whole number in µmol/L (page 39 of the KDIGO guideline).
**18. How many decimal places do you use for cystatin C concentration reporting in mg/L?**	KDIGO guideline recommends reporting cystatin C results rounded to two decimal places in mg/L (page 51 of the KDIGO guideline).
KIDGO – Kidney Disease Improving Global Outcomes. eGFR - estimated glomerular filtration rate. SRM - standard reference material. CKD-EPI - chronic kidney disease epidemiology collaboration. eGFRcrea - glomerular filtration rate estimated from serum creatinine. MDRD - modification of diet in renal disease. eGFRcys - glomerular filtration rate estimated from serum cystatin C. CAPA - Caucasian, Asian, Paediatric, Adult.	

## Discussion

We have analysed the degree of adherence of medical laboratories to KDIGO 2012 Clinical Practice Guideline for the Evaluation and Management of CKD in laboratory practice. A total of 48 out of 212 laboratories adopted the KDIGO 2012 guideline in full extent including cystatin C traceable method and eGFR. Partial KDIGO 2012 guideline implementation was found in 180 out of 212 (85%) laboratories. Biljak *et al.* performed a similar study with the purpose to improve education of laboratories and harmonization of KDIGO 2012 guideline implementation in Croatia (*10*). In our study a larger proportion of laboratories reported eGFR, the MDRD equation was less frequent, and 24 hour urine timed collection was also less frequent compared to the Croatian study. The higher proportion of MDRD equation in the study by Biljak *et al.* may be explained by the fact that their work was performed sooner after KDIGO guidelines reporting and MDRD equation preceded the CKD-EPI one. Drion *et al.* analysed national data from the Dutch external quality organization and concluded that enzymatic determination has less variability in serum creatinine measurement than Jaffe techniques, and therefore results in more accurate staging of CKD (*11*). There were 47% of laboratories with creatinine method based on non-specific Jaffé reaction in our study. We hope that this number will be decreasing in the future due to further education. The Reference Institute of Bioanalytics, German proficiency testing organization, showed in the last external quality assessment survey that 453 out of 635 (71%) laboratories used Jaffé method for creatinine determination (*12*). There was lower proportion of creatinine determination by Jaffe method in our study. Jaffe methods for the creatinine determination should be replaced by enzymatic measurement in medical laboratories.

Many patients have lost their muscle mass due to chronic conditions such as oncology disease. The study by Šálek *et al.* reported overestimation of glomerular filtration rate (GFR) by eGFRcrea compared to isotopic reference method in oncology patients before cisplatin treatment (*13*). Two laboratories did not calculate eGFRcrea in oncology patients in our study.

The KDIGO 2012 guideline recommends confirmation of CKD by estimated glomerular filtration rate from serum cystatin C (eGFRcys) when eGFRcrea is below 60 mL/min/1.73m^2^. Only 27% of laboratories in our survey measure cystatin C which enables estimation of GFR in patients with muscle loss. We plan further data analysis and investigations regarding eGFRcys. KDIGO 2012 guideline recommends CKD-EPI equation for estimation of eGFRcys with standardized cystatin C measurement. The Caucasian, Asian, Paediatric, Adult (CAPA) equation is the new alternative for estimation of GFR from standardized serum cystatin C concentration (*14*). Only a single participant used the CAPA equation in our survey. Current clinical practice relies on eGFRcrea but eGFRcys may be useful for patients who lost their muscles due to chronic diseases.

The MDRD study group which enrolled 1628 patients with CKD was used for the development of the MDRD eGFRcrea equation (*15*). The limitations of the MDRD study were that creatinine was measured by nonspecific Jaffé reaction and participants from general population were not included. Forty two laboratories in our study still used the MDRD equation. Fuček *et al.* showed that eGFR based on CKD-EPI equations correlated significantly with endemic nephropathy (*16*). Another new option for eGFRcrea is the Lund-Malmö equation (*17*). In our survey the CKD-EPI equation was used by 75% of all laboratories which were calculating eGFRcrea.

The work by Manns *et al.* demonstrated that low number of patients with CKD had albuminuria measurement. They found the care gap among all patients with CKD. Authors suggest the measurement of albumin to creatinine ratio in patients at risk for CKD development as a quality indicator (*18*). The urinary albumin to creatinine ratio is more practical than urine timed collection. A majority of laboratories in our study (92%) calculated mainly albumin to creatinine ratio from untimed urine sample compared to urine timed collection. Fung *et al*. reported that combining eGFR and albumin to creatinine ratio level was more accurate in predicting risk of cardiovascular disease and all-cause mortality. Serum creatinine with calculation of eGFRcrea and urinary albumin to creatinine ratio should be regularly monitored in diabetic patients. Early intervention to halt or even reverse the progression reduces the risk of cardiovascular disease and all-cause mortality. The findings call for more aggressive screening and intervention of albuminuria in diabetic patients (*19*). It means that all laboratories which measure urine albumin should also calculate albumin to creatinine ratio. In this survey a total of 91% (159 out of 175) of laboratories that measure urine albumin calculate also albumin to creatinine ratio. The ideal state would be that all biochemistry laboratories measure both creatinine as the marker of kidney function and urinary albumin as the marker of kidney damage. The reason is that the marker of kidney damage precedes the decline of kidney function.

The number of decimal places in reporting serum creatinine and cystatin C concentrations is the issue of uncertainty of measurement. Each series of calibrator should be accompanied with the information on its uncertainty (*20*). Kidney Disease Improving Global Outcomes 2012 guideline recommends that serum creatinine concentration should be reported and rounded to the nearest whole number when the unit of measurement is µmol/L. It is recommended to report and round serum cystatin C concentration in mg/L to two decimal places when the unit of measurement is mg/L (*21*). In this survey 59% laboratories reported serum creatinine as whole numbers and 86% reported serum cystatin C rounded to two decimal places. Biljak *et al.* reported 6 key factors for laboratories implementing the national guidelines for the diagnosis and management of CKD. The first factor is good communication between laboratory and clinicians (*22*). We also share the opinion that open and frequent communication is important for any guideline implementation.

The limitations of the study are low survey response rate and not asking about participants’ creatinine method in the survey, but obtaining it in a different way instead. Further, some participants did not respond to all questions. Information from participants may not reflect the real situation. We were not able to compare results of hospital laboratories, specialized centre laboratories, and private laboratories. In summary, a majority of laboratories in Czechia and Slovakia adopted some parts of the KDIGO 2012 guideline in laboratory practice but there is still further need of education on traceability of measurement, the importance of eGFR calculation, and harmonization of reporting of results in some cases.

## References

[1] Kidney Disease: Improving Global Outcomes (KDIGO) CKD Work Group KDIGO 2012 clinical practice guideline for the evaluation and management of chronic kidney disease. Kidney Int Suppl. 2013;3:1–150.

[2] Wang F He K Wang J Zhao MH Li Y Zhang L Prevalence and Risk Factors for CKD: A Comparison Between the Adult Populations in China and the United States. Kidney Int Rep. 2018;3:1135–43. 10.1016/j.ekir.2018.05.011 30197980 PMC6127437

[3] Brück K Stel VS Gambaro G Hallan S Völzke H Ärnlöv J CKD Prevalence Varies across the European General Population. J Am Soc Nephrol. 2016;27:2135–47. 10.1681/ASN.2015050542 26701975 PMC4926978

[4] Sikorska D Grzymislawska M Roszak M Gulbicka P Korybalska K Witowski J Simple obesity and renal function. J Physiol Pharmacol. 2017;68:175–80. 28614766

[5] Astor BC Hallan SI Miller ER 3rd Yeung E Coresh J Glomerular Filtration Rate, Albuminuria, and Risk of Cardiovascular and All-Cause Mortality in the US Population. Am J Epidemiol. 2008;167:1226–34. 10.1093/aje/kwn033 18385206

[6] Dodder NG Tai SS Sniegoski LT Zhang NF Welch MJ Certification of Creatinine in a Human Serum Reference Material by GC-MS and LC-MS. Clin Chem. 2007;53:1694–9. 10.1373/clinchem.2007.090027 17660272

[7] Grubb A Blirup-Jensen S Lindström V Schmidt C Althaus H Zegers I IFCC Working Group on Standardisation of Cystatin C (WG-SCC). First certified reference material for cystatin C in human serum ERM-DA471/IFCC. Clin Chem Lab Med. 2010;48:1619–21. 10.1515/CCLM.2010.318 21034257

[8] Catapano AL Graham I De Backer G Wiklund O Chapman MJ Drexel H 2016 ESC/EAS Guidelines for the Management of Dyslipidaemias. Eur Heart J. 2016;37:2999–3058. 10.1093/eurheartj/ehw272 27567407

[9] Zima T Racek J Tesař V Viklicky O Teplan V Schuck [Doporučeni k diagnostice chronického onemocněni ledvin (odhad glomerularni filtrace a vyšetřovani proteinurie) Česke nefrologicke společnosti ČLS JEP a Česke společnosti klinicke biochemie ČLS JEP] Klin Biochem Metab. 2014;22:138–52. [in Czech]

[10] Biljak VR Honović L Matica J Knežević B Vojak SŠ Laboratory diagnostics of chronic kidney disease in Croatia: state of the art. Biochem Med (Zagreb). 2015;25:73–83. 10.11613/BM.2015.009 25672470 PMC4401307

[11] Drion I Cobbaert C Groenier KH Weykamp C Bilo HJ Wetzels JF Clinical evaluation of analytical variations in serum creatinine measurements: why laboratories should abandon Jaffe techniques. BMC Nephrol. 2012;13:133. 10.1186/1471-2369-13-133 23043743 PMC3504563

[12] Surveys Clinical chemical analytes in serum “wet chemistry” 2019. Reference Institute of Bioanalytics. Available at: https://www.rfb.bio/cgi/displayAnaStats?rv_type=KS&rvTypeForDetails=KS&year=2019&rv_num=1&analyte=all&searchType=rv_type&anaV=7. Accessed February 15th 2019.

[13] Salek T Vesely P Bernatek J Estimated Glomerular Filtration Rate in Oncology Patients before Cisplatin Chemotherapy. Klin Onkol. 2015;28:273–7. 10.14735/amko2015273 26299741

[14] Grubb A Horio M Hansson L-O Björk J Nyman U Flodin M Generation of a New Cystatin C–Based Estimating Equation for Glomerular Filtration Rate by Use of 7 Assays Standardized to the International Calibrator. Clin Chem. 2014;60:974–86. 10.1373/clinchem.2013.220707 24829272

[15] Levey AS Bosch JP Lewis JB Greene T Rogers N Roth D A more accurate method to estimate glomerular filtration rate from serum creatinine: a new prediction equation. Modification of Diet in Renal Disease Study Group. Ann Intern Med. 1999;130:461–70. 10.7326/0003-4819-130-6-199903160-00002 10075613

[16] Fuček M Dika Ž Karanović S Vuković Brinar I Premužić V Kos J Reliability of CKD-EPI predictive equation in estimating chronic kidney disease prevalence in the Croatian endemic nephropathy area. Biochem Med (Zagreb). 2018;28:010701. 10.11613/BM.2018.010701 29187794 PMC5701772

[17] Björk J Bäck SE Sterner G Carlson J Lindstrom V Bakoush O Prediction of relative glomerular filtration rate in adults: new improved equations based on Swedish Caucasians and standardized plasma-creatinine assays. Scand J Clin Lab Invest. 2007;67:678–95. 10.1080/00365510701326891 17852799

[18] Manns L Scott-Douglas N Tonelli M Weaver R Tam-Tham H Chong C A Population-Based Analysis of Quality Indicators in CKD. Clin J Am Soc Nephrol. 2017;12:727–33. 10.2215/CJN.08720816 28377473 PMC5477213

[19] Fung CS Wan EY Chan AK Lam CL Association of estimated glomerular filtration rate and urine albumin-to-creatinine ratio with incidence of cardiovascular diseases and mortality in chinese patients with type 2 diabetes mellitus - a population-based retrospective cohort study. BMC Nephrol. 2017;18:47. 10.1186/s12882-017-0468-y 28152985 PMC5290675

[20] Ćelap I Vukasović I Juričić G Šimundić AM Minimum requirements for the estimation of measurement uncertainty: Recommendations of the joint Working group for uncertainty of measurement of the CSMBLM and CCMB. Biochem Med (Zagreb). 2017;27:030502. htps://. 10.11613/BM.2017.030502 29180913 PMC5696748

[21] The New International Recommendations for Chronic Kidney Disease. Available at: https://www.aacc.org/publications/cln/articles/2014/october/kidney-disease. Accessed January 16th 2019.

[22] Biljak VR Honović L Matica J Krešić B Vojak SŠ The role of laboratory testing in detection and classification of chronic kidney disease: national recommendations. Biochem Med (Zagreb). 2017;27:153–76. 10.11613/BM.2017.019 28392738 PMC5382859

