# Corrigendum to: Assessment of the degree of adherence of medical laboratories to KDIGO 2012 guideline for evaluation and management of CKD in Czechia and Slovakia

**DOI:** 10.11613/BM.2019.031202

**Published:** 2019-10-15

**Authors:** Tomáš Šálek, Bedřich Friedecký, Josef Kratochvíla, Květa Pelinková, Marek Budina

**Affiliations:** 1 Department of Clinical Biochemistry and Pharmacology, Tomas Bata Hospital, Zlín, Czech Republic; 2 Department of Biomedical Sciences, Medical Faculty of the University of Ostrava, Ostrava - Zábřeh, Czech Republic; 3 SEKK, spol. s.r.o., Pardubice, Czech Republic; 4 Institute of Medical Biochemistry and Laboratory Diagnostics, General University Hospital and The First Faculty of Medicine of Charles University in Prague, Prague, Czech Republic

This is a correction of Biochem Med (Zagreb)2019;29(3):030704. DOI: https://doi.org/10.11613/BM.2019.030704

Since the publication of the article, the authors have noticed that their first names and surnames in the byline were listed in reverse. The correct byline is presented above. We apologize to the authors for any inconvenience caused to the readers.

